# Antimicrobial peptide GH12 suppresses cariogenic virulence factors of *Streptococcus mutans*

**DOI:** 10.1080/20002297.2018.1442089

**Published:** 2018-02-26

**Authors:** Yufei Wang, Xiuqing Wang, Wentao Jiang, Kun Wang, Junyuan Luo, Wei Li, Xuedong Zhou, Linglin Zhang

**Affiliations:** ^a^ State Key Laboratory of Oral Diseases & National Clinical Research Centre for Oral Disease, Department of Cariology and Endodontics West China Hospital of Stomatology, Sichuan University, Chengdu, China

**Keywords:** Dental caries, antimicrobial cationic peptides, anti-bacterial agents, acidogenicity, aciduricity, biofilms

## Abstract

Cariogenic virulence factors of *Streptococcus mutans* include acidogenicity, aciduricity, and extracellular polysaccharides (EPS) synthesis. The *de novo* designed antimicrobial peptide GH12 has shown bactericidal effects on *S. mutans*, but its interaction with virulence and regulatory systems of *S. mutans* remains to be elucidated. The objectives were to investigate the effects of GH12 on virulence factors of *S. mutans*, and further explore the function mechanisms at enzymatic and transcriptional levels. To avoid decrease in bacterial viability, we limited GH12 to subinhibitory levels. We evaluated effects of GH12 on acidogenicity of *S. mutans* by pH drop, lactic acid measurement and lactate dehydrogenase (LDH) assay, on aciduricity through survival rate at pH 5.0 and F_1_F_0_-ATPase assay, and on EPS synthesis using quantitative measurement, morphology observation, vertical distribution analyses and biomass calculation. Afterwards, we conducted quantitative real-time PCR to acquire the expression profile of related genes. GH12 at 1/2 MIC (4 mg/L) inhibited acid production, survival rate, EPS synthesis, and biofilm formation. The enzymatic activity of LDH and F_1_F_0_-ATPase was inhibited, and *ldh, gtfBCD, vicR, liaR*, and *comDE* genes were significantly downregulated. In conclusion, GH12 inhibited virulence factors of *S. mutans*, through reducing the activity of related enzymes, downregulating virulence genes, and inactivating specific regulatory systems.

## Introduction

Dental caries is a transmissible infectious disease caused by cariogenic bacteria, and *Streptococcus mutans* has been recognized as the principal etiological agent of dental caries [1]. Cariogenic virulence factors of *S. mutans* lead to abundant acid production, acid tolerance and stable biofilm formation [2]. Production of acid during metabolism of carbohydrates (acidogenicity) is one of the main virulence factors of *S. mutans*, in which lactate dehydrogenase (LDH) plays a critical role [3]. Aciduricity is also a pivotal virulence factor of *S. mutans*. Membrane-bound F_1_F_0_-ATPase works as a pump to transport protons from cells and to maintain internal pH values. This is believed as the determinant of aciduricity of this bacterium [4]. Another critical virulence factor of *S. mutans* is its ability to produce glucosyltransferases (GTFs) to catalyze synthesis of intracellular polysaccharides (IPS) and extracellular polysaccharides (EPS) from sucrose [5]. EPS, especially water-insoluble glucans, significantly contribute to the biofilm formation and structural integrity [6]. Hence, suppression of cariogenic virulence of *S. mutans* could be an appealing approach to preventing dental caries.

However, the cariogenic potential of *S. mutans* is not the consequences of independent expression by several virulence genes, but the results of series of coordinate regulatory events. Regulatory systems are involved in enhancing the ecological fitness and cariogenic virulence of *S. mutans* [7]. The VicRKX and LiaFSR two-component signal transduction systems (TCSTS) have been reported as the master regulatory system involved in expression of GTFs, biofilm formation and environment stress tolerance in *S. mutans* [8,9]. The ComCDE system quorum sensing (QS) system enables intra-species cell–cell communication [10]. As these TCSTS are selective for the bacteria and not the host, they are regarded as excellent therapeutic targets for antimicrobial agents [11].

In recent years, studies about antimicrobial peptides (AMPs) have been extensively conducted around the world [12]. Over the last decade, a significant leap in studies about control of oral pathogens by AMPs was observed [13]. So far, many natural or synthetic AMPs have shown inhibitory effects on growth of planktonic cells and biofilm of *S. mutans in vitro*, including defensins, Cathelicidin LL-37, Histatin 5, Human Lactoferrin [14], KSL [15], L-K6 [16], Bac8c [17], and C16G2 [18]. Furthermore, the placebo-controlled clinical data of C16G2 rinse showed this AMP was safe and reduced *S. mutans* and enamel demineralization [19]. These findings suggest that AMPs have potential to be another safe and promising solution to dental caries. In combat against cariogenic bacteria, our group *de novo* designed and synthesized a series of cationic, amphipathic α-helical AMPs and selected the most promising one, GH12, a 12-amino acids peptide with optimal structure and potency. Previous studies [20,21] showed that GH12 at the concentration of 8 mg/L, the minimal inhibitory concentration (MIC) and minimal bactericidal inhibitory concentration (MBC) of GH12 against *S. mutans*, was potent against growth of planktonic bacteria and had a rapid mechanism of action, killing 90% of bacteria in less than 5 min of exposure. Moreover, GH12 effectively inhibited *S. mutan*s biofilm formation and metabolism, as well as significantly reduced the biomass of 1-day old *S. mutans* biofilm. Cytomembrane observation showed that GH12 had a mechanism of killing *S. mutans* through formation lysis and pores on the cell membrane and envelope. Meanwhile, GH12 had showed little toxic effect on the viability of human gingival fibroblasts [21], and kept stable in human saliva [20].

GH12 has shown anticaries potential because of its bactericidal effects on *S. mutans*, but its interaction with virulence factors and regulatory systems of *S. mutans* remains to be elucidated. The objectives of this study were to investigate effects of GH12 on acid production, acid tolerance, EPS synthesis, and biofilm formation of *S. mutans in vitro*, and then further explore the function mechanism of GH12 at enzymatic and transcriptional levels. To avoid decrease in bacterial population and viability of *S. mutans*, we limited GH12 to subinhibitory levels. It is the first time to systematically investigate effects of AMPs on virulence and regulatory systems of *S. mutans*. Thereby, more information about GH12 could be obtained to further explain its anticaries potential.

## Materials and methods

### Peptides, bacterial strains, and growth conditions

Peptide GH12 (Gly-Leu-Leu-Trp-His-Leu-Leu-His-His-Leu-Leu-His-NH_2_) was synthesized, identified, and purified to 98% by GL Biochem (Shanghai, China) as described before [21]. The peptide was dissolved in sterile deionized water and stored at −20°C. All chemicals and assay kits were purchased from Sigma-Aldrich (St. Louis, MO) unless otherwise stated. *S. mutans* UA159 was obtained from the State Key Laboratory of Oral Diseases at Sichuan University (Chengdu, China) and grown in brain–heart infusion broth (BHI; Oxoid, Basingstoke, Hampshire, UK) anaerobically (85% N_2_, 10% H_2_ and 5% CO_2_) at 37°C [22]. For lactic acid measurement, buffered peptone water (BPW, Nissui, Tokyo, Japan) was used [23], and for acid tolerance assays, tryptone-yeast extract medium containing 20 mM glucose (TYEG) was applied [24].

### Growth curve assay

Aliquots of overnight culture of *S. mutans* were diluted in BHI broth to obtain the final concentration of 1 × 10^7^ CFU/mL. GH12 was then added into tubes filled with *S. mutans* culture to final concentrations of 1/8 MIC (1 mg/L), 1/4 MIC (2 mg/L) and 1/2 MIC (4 mg/L). Sterile deionized water acted as vehicle control. These tubes were incubated at 37°C anaerobically for 24 h. The absorbance at 600 nm (A_600_) was determined using a microplate spectrophotometer (Multiskan GO; Thermo Scientific, Waltham, MA) every hour throughout 24 h of incubation. The experiments were repeated three times independently.

### MTT assay

The effects of GH12 at sub-MIC levels on the viability of *S. mutans* was assessed by the 3-(4, 5-dimethylthiazol-2-yl)-2, 5-diphenyltetrazolium bromide (MTT) staining method as described [25,26] with some modification. Briefly, *S. mutans* was suspended with BHI broth containing GH12 at sub-MIC levels or sterile deionized water. Volumes of 200 μL of different suspension were inoculated into wells in a 96-well U-bottom microtiter plate, and incubated at 37°C for 24 h. After centrifugation (4,500 g, 5 min, 4°C), the supernatant was decanted. 200 μL of MTT dye (500 mg/L MTT in PBS) was added into the wells. Following incubation at 37°C for 2 h, the MTT solution was replaced with 200 μL DMSO to dissolve the formazan crystals. The absorbance at 540 nm (A_540_) was measured. The experiments were repeated for three times independently.

### Glycolytic pH drop assay

The effect of GH12 on *S. mutans* glycolysis was measured as described elsewhere with some modifications [27]. Briefly, *S. mutans* was harvested at mid-logarithmic phase, washed with salt solution (50 mM KCl + 1 mM MgCl_2_), and resuspended in a salt solution containing GH12 at sub-MIC levels or sterile deionized water. Initial concentration of *S. mutans* was adjusted to 1 × 10^7^ CFU/mL. Glucose was added to a final concentration of 1% (w/v) and the initial pH of the mixtures was then adjusted to 7.2. The decrease in pH was monitored over a period of 120 min by Orion Dual Star, pH/ISE Benchtop (Thermo Scientific, Waltham, MA). The experiments were repeated for three times independently.

### Lactic acid measurement

For lactic acid measurement, the cells of *S. mutans* were harvested, washed twice with phosphate buffered saline (PBS) and resuspended to a final concentration of 1 × 10^7^ CFU/mL in a 24-well plate with 1.5 mL BPW supplemented with 0.2% sucrose containing sub-MIC levels of GH12 in each well. The vehicle control contained no GH12. The plate was further incubated for 120 min at 37°C anaerobically. After removing planktonic cells by centrifugation (8,000 g, 5 min, 4°C), the supernatants were decanted to measure lactate concentrations according to the manuscript of the Lactate Assay Kit (MAK064). The absorbance at 570 nm (A_570_) was recorded using a microplate spectrophotometer and lactate concentrations were calculated using standard curves. The experiments were repeated for three times independently.

### LDH assay

The crude LDH was extracted according to Xu et al. [28]. The crude LDH was then treated with GH12 at sub-MIC levels for 30 min. The activity of LDH was estimated using the LDH Activity Assay Kit (MAK066). According to the technical bulletin of this assay kit, the absorbance at 450 nm at initial time (A_450initial_) and endpoint (A_450final_) was recorded, and then ΔA_450_ was calculated to quantify the enzymatic activity. The results were expressed as the percentage of ΔA_450_ relative to that of the untreated control. The experiments were repeated for five times independently.

### Acid tolerance assay

The effect of GH12 on the acid tolerance of *S. mutans* was evaluated by measuring the viability of bacteria after 120 min exposure of pH 5.0 [24]. *S. mutans* was grown in TYEG broth until mid-logarithmic phase. After centrifugation, the cells were resuspended (1 × 10^7^ CFU/mL) in TYEG broth buffered with 40 mM phosphate/citrate buffer (pH 5.0) containing sub-MIC levels of GH12, and incubated at 37°C for 2 h. The control mixture contained no GH12. Samples were removed before and after incubation at pH 5.0 for viable counts. The experiments were repeated for three times independently.

### Proton permeability and F_1_F_0_-atpase assays

*S. mutans* was permeabilized according to the method described by Belli et al. [27]. Then, permeabilized cells were pretreated with sub-MIC levels of GH12 for 15 min. The F_1_F_0_-ATPase activity was determined by the amount of released inorganic phosphate in the reaction mixture of 75 μL of permeabilized cells and 3 mL of 50 mM Tris-maleate buffer (pH 6.0) with 10 mM MgSO_4_. When the mixture was heated to 37°C, the reaction was initiated by adding 30 μL of 0.5 M ATP (pH 6.0). After 30 min of reaction, the released phosphate was determined [29], and the results were expressed as enzymatic activity relative to that of the untreated control. The experiments were repeated for five times independently.

### Biofilm formation assay

The effect of GH12 at sub-MIC levels on biofilm formation was evaluated as described before [21]. Briefly, *S. mutans* was diluted with BHI broth supplemented with 1% sucrose (BHIS) and GH12 at sub-MIC levels to a final concentration of 1 × 10^6^ CFU/mL. After anaerobic incubation (24 h, 37°C), culture supernatants and planktonic cells were removed. After fixing the biofilms with methanol for 15 min and staining them with 0.1% (w/v) crystal violet for 5 min, the dye bound to the cells was resolubilized with 33% (v/v) glacial acetic acid. The absorbance at 595 nm (A_595_) was measured to quantify biofilm formation and the results were expressed as the percentage of A_595_ relative to that of the untreated control. The experiments were repeated for three times independently.

### Water-insoluble EPS measurement

Biofilms of *S. mutans* were produced with different sub-MIC levels of GH12 in 2 mL BHIS in 24-well plates. The water-insoluble EPS of biofilms was determined by the anthrone method [30]. Briefly, biofilms were collected by sonication/vortexing in PBS buffer. Then the precipitate was obtained by centrifugation, washed twice with sterile water and resuspended in 4 mL of 0.4 M NaOH. After centrifugation, 200 μL of supernatant was mixed with 600 μL of anthrone reagent at 95°C for 6 min. The absorbance at 625 nm (A_625_) was monitored and the concentrations of water-insoluble EPS were calculated using standard curves. The experiments were repeated for three times independently.

### Confocal laser scanning microscope (CLSM) observation

For EPS staining, biofilms of *S. mutans* were produced on glass coverslips in a 24-well plate under the same conditions mentioned before. 2.5 μM SYTO®9 (S34854; Molecular Probes™, Invitrogen, Carlsbad, CA) and 2.5 μM Alexa Fluor® 647 (D22914; Molecular Probes™) were used according to the manufacturer (Invitrogen). Alexa Fluor® 647-labelled dextran conjugate was added at the beginning of biofilm formation, and 50 μL of SYTO®9 was used to stain bacteria after biofilm formation. The biofilms were imaged with a Leica DMIRE2 confocal laser scanning microscope (Leica, Wetzlar, Germany) equipped with a 60 × oil immersion objective lens. The image channels were set according to the manufacturer. Each biofilm was scanned at five randomly selected positions. All three-dimensional reconstructions of biofilms were performed with Imaris 7.0.0 (Bitplane, Zürich, Switzerland). The vertical distribution analyses were performed with LAS AF Lite (Leica), and the calculation of bacteria/EPS biomass was performed with COMSTAT (http://www.imageanalysis.dk) [31].

### Quantitative real-time PCR

To evaluate the effect of GH12 on associated gene expressions, *S. mutans* was grown in BHI broth supplemented with sub-MIC levels of GH12 until late exponential phase. The control group was not treated with GH12. The RNA isolation and purification were conducted as described before [28]. First-strand cDNAs were synthesized using PrimeScript™ RT reagent Kit with gDNA Eraser (RR047A; Takara Bio, Shiga, Japan), according to the manufacturer. Tested genes and specific primers were listed in Table 1 [32–34]. Each PCR reagent (25 μL) contained SYBR® Premix Ex Taq ™ II (RR820A; Takara Bio), cDNA samples (80 ng) and forward and reverse gene-specific primers (10 μM, 1 μL each). The qPCR was performed on CFX96 Real-Time System (C1000™ Thermal Cycler; Bio-Rad, Hercules, CA) using the same thermocycling conditions as in a previous study [35]. The 2^−ΔΔCt^ method was used to calculate gene expression fold change, and different gene expressions were normalized to the levels of 16S rRNA gene transcripts. The experiments were repeated for three times independently.

**Table 1.** Specific primers of quantitative real-time PCR.

Primers	Sequence (F and R)	References
16S RNA	AGCGTTGTCCGGATTTATTG	[32]
	CTACGCATTTCACCGCTACA	
*ldh*	AAAAACCAGGCGAAACTCGC	[33]
	CTGAACGCGCATCAACATCA	
*atpD*	TGTTGATGGTCTGGGTGAAA	[28]
	TTTGACGGTCTCCGATAACC	
*gtfB*	CACTATCGGCGGTTACGAAT	[32]
	CAATTTGGAGCAAGTCAGCA	
*gtfC*	GATGCTGCAAACTTCGAACA	[32]
	TATTGACGCTGCGTTTCTTG	
*gtfD*	TTGACGGTGTTCGTGTTGAT	[32]
	AAAGCGATAGGCGCAGTTTA	
*vicR*	CGTGTAAAAGCGCATCTTCG	[33]
	AATGTTCACGCGTCATCACC	
*liaR*	CATGAAGATTTAACAGCGCG	[33]
	CGTCCTGTGGCACTAAATGA	
*comD*	TTCCTGCAAACTCGATCATATAGG	[34]
	TGCCAGTTCTGACTTGTTTAGGC	
*comE*	TTCCTCTGATTGACCATTCTTCTG	[34]
	GAGTTTATGCCCCTCACTTTTCAG	

### Statistics analysis

Differences between the experimental group and the untreated control group were compared using one-way ANOVA and Tukey HSD tests. Statistical analyses were performed using SPSS 20.0 (IBM, Chicago, IL) at a significance level of 0.05.

## Results

### GH12 at sub-MIC levels is not interfering with the basic viability of planktonic S. mutans

**Figure 1.**
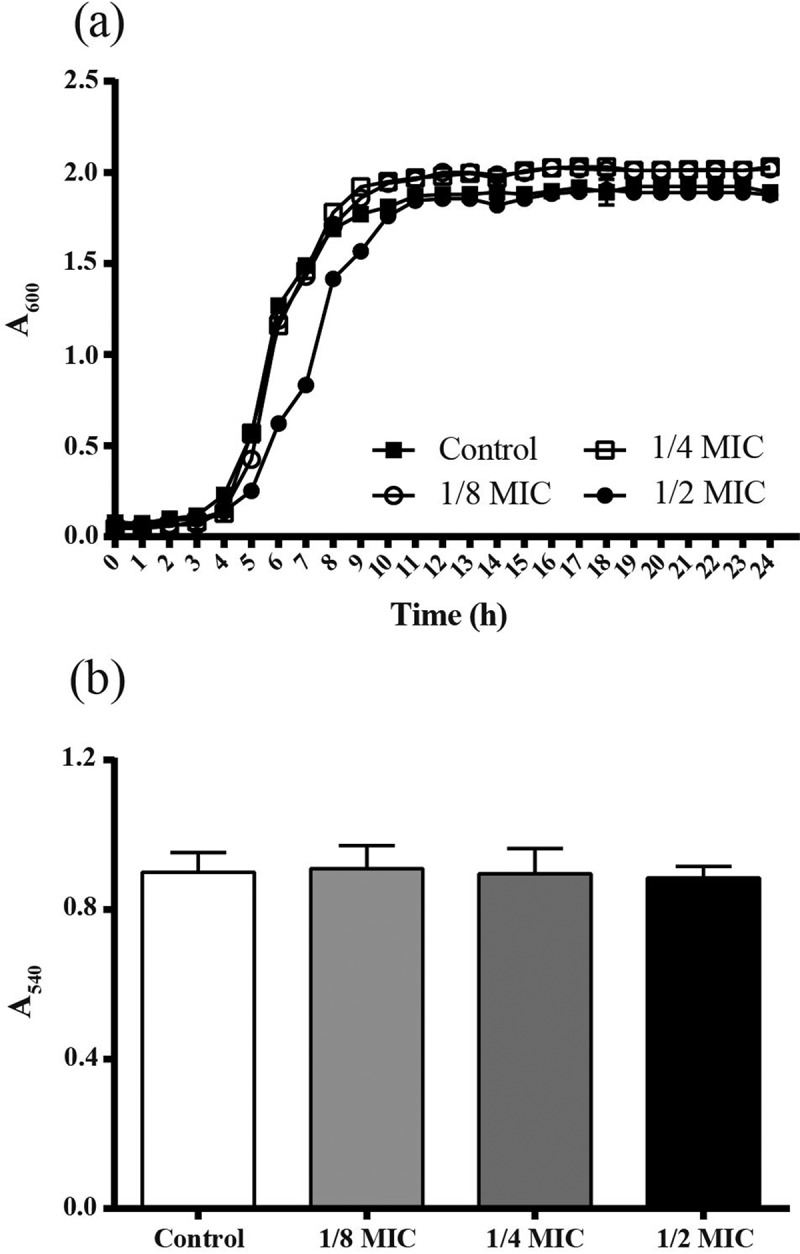
Effects of GH12 at sub-MIC levels on (a) the growth curves and (b) MTT metabolic activity of *S. mutans* in the absence and presence of GH12 at sub-MIC levels. Data are presented as means ± standard deviations.

We evaluated the effects of GH12 at sub-MIC levels on basic viability of *S. mutans* by growth curves and MTT assay. As shown in Figure 1(a), when treated by GH12 at 1/4 MIC and 1/8 MIC, there was no significant alternation in the growth pattern of *S. mutans*. When treated by GH12 at 1/2 MIC, *S. mutans* exhibited an extended lag phase and lower absorbance during the logarithmic phase. At the end of 24 h, the mean absorbance of 1/2 MIC group did not significant differ from those of the non-treated control (*P* > 0.05). The outcome of MTT assay (Figure 1(b)) also revealed that there was no significant difference in cell viability of the control and treated *S. mutans* within 24 h (*P* > 0.05).

### GH12 inhibits the acidogenicity of S. mutans *in vitro*

**Figure 2.**
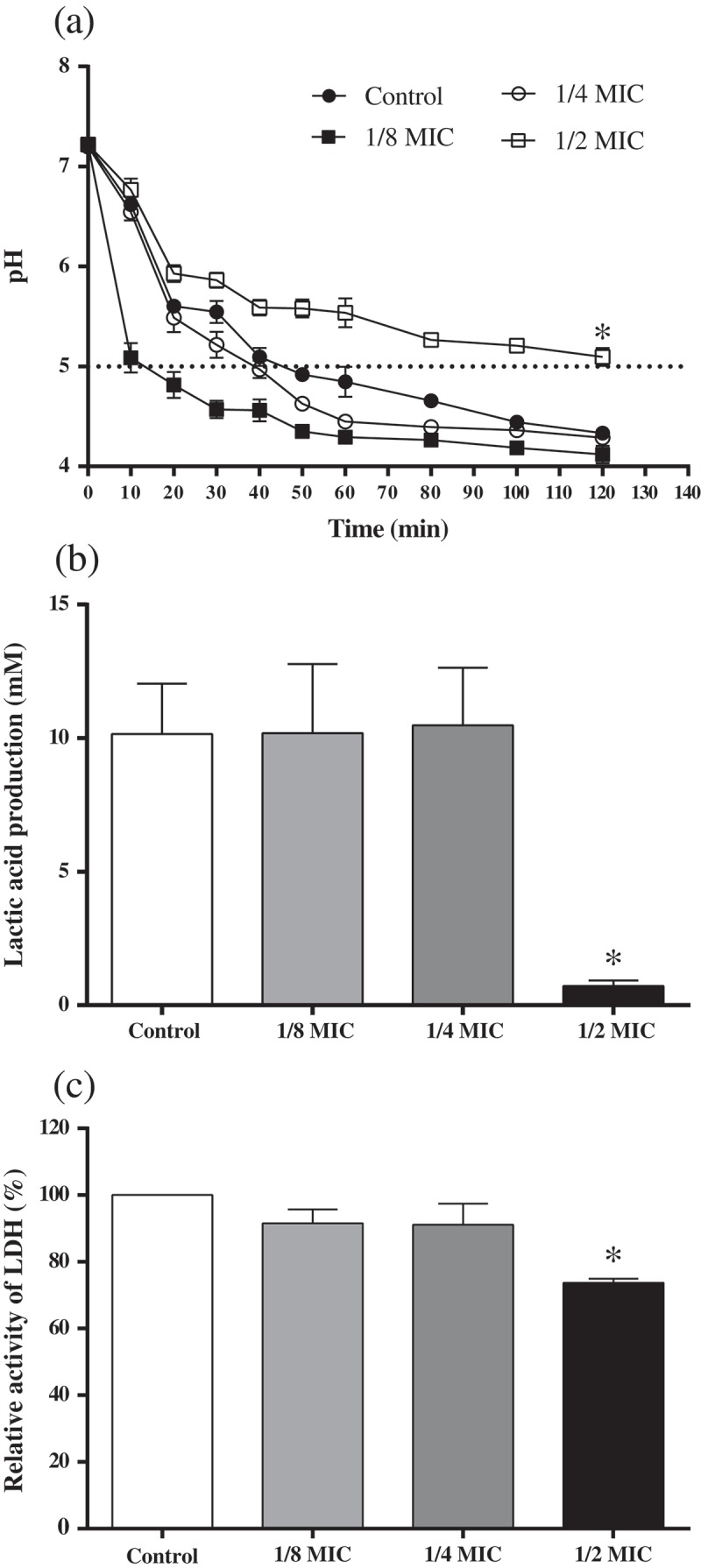
Effects of GH12 on glycolytic pH drop (a), lactic acid production (b), and lactate dehydrogenase (LDH) activity (c) of S. mutans. Horizontal dotted line represents the lethal pH value (pH 5.0) to S. mutans. Data are presented as means ± standard deviations. *P < 0.05: significantly different from the vehicle control (sterile deionized water).

We determined the effects of GH12 at sub-MIC levels on acidogenicity by monitoring the glycolytic pH drop, lactic acid production, and LDH activity of the *S. mutans* culture. As shown in Figure 2(a), when the *S. mutans* culture was treated with GH12 at 1/2 MIC, its glycolytic pH dropped slower, and the terminal pH was significantly higher versus that of control (*P* < 0.05). However, the terminal pH values were not significantly affected by GH12 at 1/4 MIC and 1/8MIC (*P* > 0.05). Further assays showed that 1/2 MIC of GH12 suppressed the lactic acid production (Figure 2(b)), and LDH activity (Figure 2(c)) of *S. mutans* cells respectively, which were consistent with the results of the glycolytic pH drop assay.

### GH12 inhibits the aciduricity of S. mutans *in vitro*

**Figure 3.**
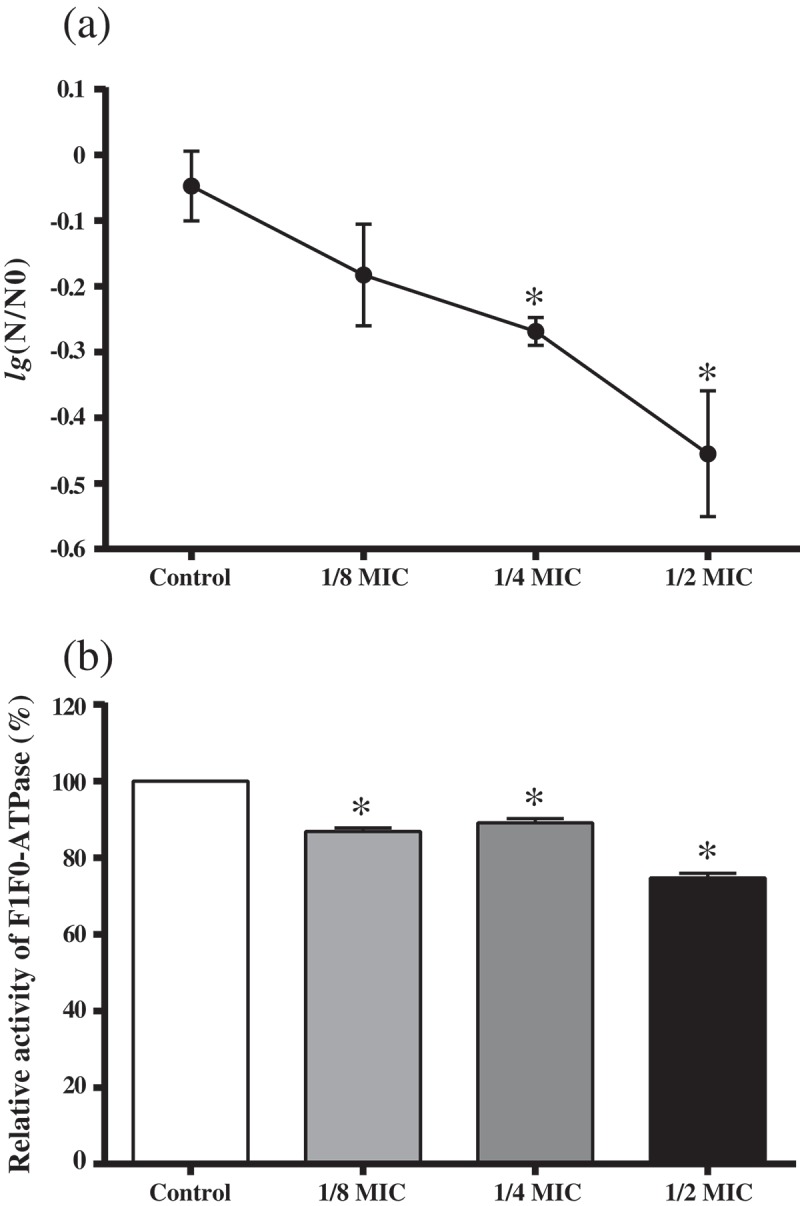
Effects of GH12 on aciduricity of *S. mutans*. (a) The survival rate of *S. mutans* at pH 5.0. *N*_0_ and *N* represent CFU counts before and after 2 h treatment in pH 5.0 culture, respectively. (b) Relative activity of F_1_F_0_-ATPase of *S. mutans*. Data are presented as means ± standard deviations. *P < 0.05: significantly different from the vehicle control (sterile deionized water).

The acidurity of *S. mutans* was also inhibited by GH12 at sub-MIC levels. Firstly, Figure 3(a) showed that the survival rate of *S. mutans* at pH 5.0 was significantly decreased in the presence of GH12 at 1/2 MIC and 1/4 MIC (*P* < 0.05). At the same time, GH12 exhibited inhibitory effects on the activity of the F_1_F_0_-ATPase of *S. mutans. The* F_1_F_0_-ATPase activity was reduced by 25.26% at 1/2 MIC of GH12 (Figure 3(b)).

### GH12 inhibits water-insoluble EPS synthesis and biofilm formation of S. mutans *in vitro*

**Figure 4.**
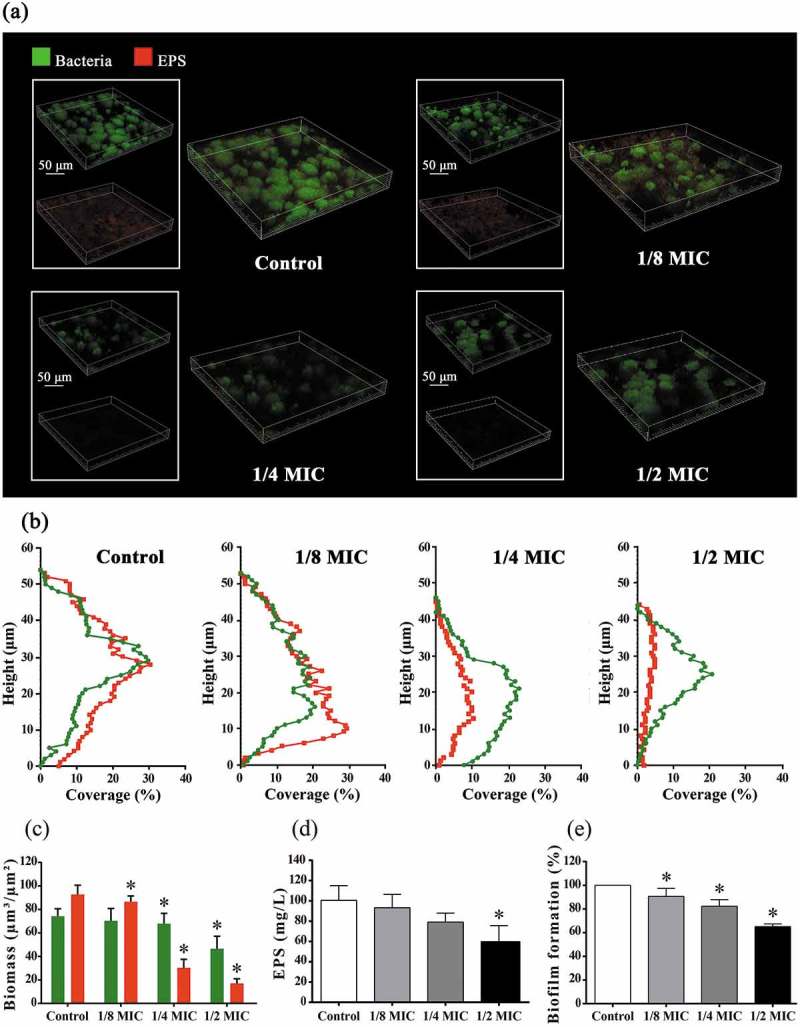
Effects of GH12 on polysaccharides synthesis and biofilm of *S. mutans*. (a) Three-dimensional CLSM image *S. mutans* biofilm (bacteria, stained green; EPS, stained red). (b) Vertical distribution of bacteria and EPS calculated from CLSM imaging data sets. (c) The biomass of EPS and bacteria, calculated according to five random sights of biofilms by COMSTATA. (d) Quantitative data of the water-insoluble EPS amount of *S. mutans* biofilms measured by the anthrone method. (e) Quantitative data of the *S. mutans* biofilm formation measured by crystal violet dye. Data are presented as means ± standard deviations. *P < 0.05: significantly different from the vehicle control (sterile deionized water).

GH12 at sub-MIC levels disrupted the ability of *S. mutans* to synthesize water-insoluble EPS and form biofilm. As shown in Figure 4(a), bacteria in *S. mutans* biofilms were labelled green, and EPS were labelled red. Compared with biofilm of the untreated control, biofilms treated with GH12 at sub-MIC levels became thinner and looser, their green area was reduced and their red area was diminished. Figure 4(b) further demonstrated that GH12 at 1/4 and 1/2 MIC markedly decreased the height of biofilms, and reduced the coverage of bacteria and EPS at the same time. Consequently, treatment with GH12 at 1/4 and 1/2 MIC resulted in significant reduced biomass of bacteria and EPS matrix (Figure 4(c)). These findings were confirmed by quantitative analyses of EPS and biofilm formation. As shown in Figure 4(d), 1/2 MIC of GH12 signally reduced the amount of water-insoluble EPS (*P* < 0.05). Figure 4(e) showed that GH12 inhibited biofilm formation in a dose-dependent manner, and there was a 34.88% reduction in biofilm formation in 1/2 MIC group compared with the control group (*P* < 0.05).

### GH12 inhibits expression of virulence genes and TCSTS genes of S. mutans *in vitro*

**Figure 5.**
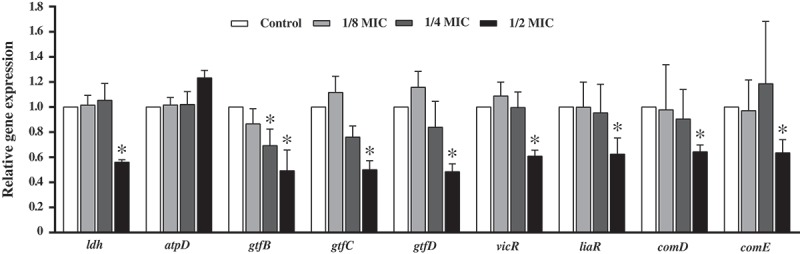
Expression profile of various virulence genes and TCSTS genes of *S. mutans* in response to the treatment with GH12. Gene expression was quantified by real-time PCR, with 16S rRNA as an internal control. Data are presented as means ± standard deviations. *P < 0.05: significantly different from the vehicle control (sterile deionized water).

The expression profiles of various virulence genes (*ldh, atpD, gtfB, gtfC, gtfD*) and TCSTS genes (*vicR, liaR, comD, comE*) of *S. mutans* treated with GH12 at sub-MIC levels wre determined (Figure 5). Almost the entire set of genes was significantly downregulated by 1/2 MIC of GH12 (*P *< 0.05), except for *atpD*, which was upregulated but not significantly (*P *> 0.05). Notably, all GTF-associated genes (*gtfB, gtfC, gtfD*) were also downregulated by 1/4 MIC of GH12, but only *gtfB* exhibited significant differences (*P* < 0.05).

## Discussion

Previously, we found that the classic *de novo* synthetic antimicrobial decapeptide KSL shows low potency against cariogenic bacteria, with a MIC of 62.5 mg/L and MBC of 125 mg/L against *S. mutans* and *L. acidophilus* [15]. According to structure/activity relationship studies by Giangaspero et al. [36], we concluded that this poor efficacy may be related to that KSL does not adopt the optimal structure of cationic, amphipathic α-helical AMPs. Similarly, we analysed the sequence and structure of a 13-residue AMP L-K6, whose hydrophilic sector was also not clearly separate from the hydrophobic sector [16]. Thus, our strategy is to design and synthesize *de novo* a series of cationic AMPs that would be shorter, and adopt optimal amphipathic α-helical structure. Designed through integration of existing design strategies, GH12 owns the optimal net positive charge, content of hydrophobic residues and relative amphipathicity, adopts >80% the α-helical composition and shows superior antimicrobial activity against *S. mutans* with MIC and MBC of 8 mg/L [21]. Notably, Shang et al. [16] deduced that L-K6 exerts bactericidal rather than bacteriostatic activity against *S. mutans*, due to that the MBC values (12.5 μM, 19.40 mg/L) of L-K6 against *S. mutans* were four-fold higher than the MIC values. However, the MIC and MBC of GH12 against *S. mutans* were very close, suggesting that GH12 may have other potentials.

It is well documented that the virulence factors of *S. mutans* essentially attribute to its clinical complications [37]. Nevertheless, up til now, for AMPs, there has been little literature about their anti-virulence activities against *S. mutans*. Prior to our study, only that on Bac8c [17] reported limited information on its inhibition of the GTF gene expression at two-fold MBC against *S. mutans*. Thus, to our knowledge, this is the first time to systematically investigate effects of AMPs on virulence and regulatory systems of *S. mutans*. In the present study, we found that GH12 at 1/2 MIC inhibited various cariogenic virulence factors of *S. mutans*, resulting in reduced acidogenicity, compromised aciduricity, declined EPS synthesis and defective ability to form biofilms. We also proved that GH12 functioned at enzymatic and transcriptional levels.

Glycolysis is the main pathway to produce acid in *S. mutans*. LDH is one of the most important enzymes in the process. Deficiency of LDH implies that *S. mutans* lose cariogenic potential [38,39]. The results from the glycolysis pH drop showed reduction in the initial rate of pH drop and rise in terminal pH caused by 1/2 MIC of GH12, which were consistent with results of the lactic acid measurement. These findings suggest the impairment in acidogenicity of *S. mutans*. Moreover, GH12 at 1/2 MIC suppressed the LDH at both the enzymatic and transcriptional levels *in vitro*. F_1_F_0_-ATPase, plays a major role in maintenance of ΔpH across the cell membrane of *S. mutans*, and the positive correlation between ATPase levels and acid tolerance has been proved [27,40]. GH12 at 1/2 MIC showed remarkable reduction in activity of the F_1_F_0_-ATPase, which may directly lead to a rise in the cytoplasmic acidity, followed by mortal disruption of physiological processes in *S. mutans*. Thus, the survival rate of *S. mutans* at pH 5.0 was significantly reduced by GH12 at 1/2 MIC. But the expression of *atpD*, a gene encoding α subunit of F_1_F_0_-ATPase [41], was upregulated although not statistically significant. Considering that AMPs GH12 function via the mechanism of accumulating at the bacterial membrane and inserting into lipid bilayers [21,42,43], we speculate that GH12 may directly affect the activity of F_1_F_0_-ATPase, which is located at cell membrane as well. Previous studies implied that the ATPase activity does not necessarily correlate with transcription or translation levels of associated genes [44]. The action of GTFs (GTFB, GTFC, and GTFD) is recognized as the major mechanism behind sucrose-dependent adhesion and biofilm formation. GTFB (encoded by *gtfB*) produces highly insoluble glucans, which constitute the scaffold of the EPS matrix. GTFC (encoded by *gtfC*) catalyzes the formation of a mixture of soluble and insoluble glucans, which shape the initial EPS layers and provide binding sites for *S. mutans* [1,2,45]. In this study, GH12 at 1/2 MIC exhibited a strong ability to inhibit the synthesis of water-insoluble EPS. CLSM observation demonstrated that GH12 at 1/2 MIC disrupted the structure and integrity of *S. mutans* biofilm. Results of quantitative PCR confirmed that these inhibitory effects were due to the significant suppression of GTFs at the transcriptional level. Notably, GH12 at 1/4 MIC just inhibited the gene expression of *gtfB* significantly (*P* < 0.05), but it also showed strong capability to inhibit EPS synthesis and biofilm formation, which may results from that *gtfB* and *gtfC* genes are in an operon-like arrangement [46].

**Figure 6.**
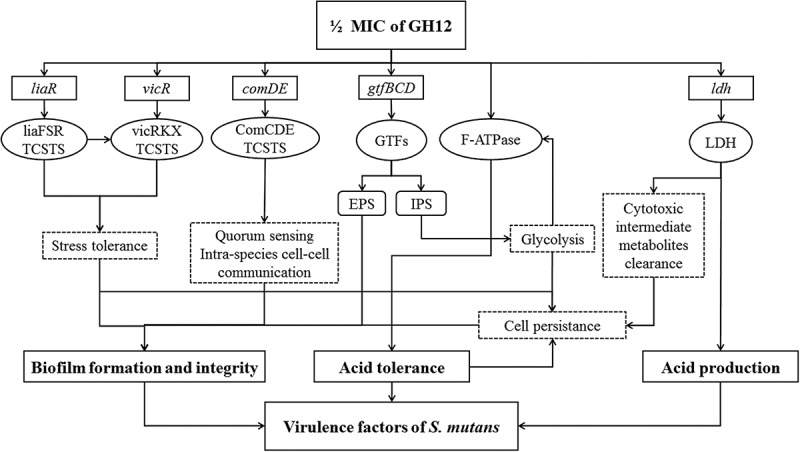
Inhibitory pathways of GH12 at 1/2 MIC on virulence factors of *S. mutans*, through regulating various genes and enzymes involved in *S. mutans* cariogenic virulence.

To cope with various environmental stress, *S. mutans* has developed several regulatory systems. *vicR* gene encodes a VicR response regulator, which is a necessary part of VicRKX TCSTS [8]. It has been reported that VicRKX TCSTS have the ability to influence the ComCDE system and gene expression of *gtfBCD* [8]. The *liaR* gene products play an important role in biofilm formation and stress response of *S. mutans* [47]. Moreover, phosphotransfering from LiaS to LiaR is necessary to promote expression of *vicRKX* genes [48]. Thus, the downregulation of *vicR* and *liaR* may result from not only GH12 treatment, but also the interaction between the two genes. *comD* encodes the histidine kinase receptor to response to competence-stimulating peptide (CSP), and *comE* encodes an intracellular response regulator to mediate expressions of downstream genes. Activated ComE enables the production of mutacin Ⅳ and Ⅴ, as well as genetic competence [49]. Significant downregulation of *comDE* was also observed in the 1/2 MIC group, and downregulation of *comDE* by 1/2 MIC of GH12 suppresses regulation of genetic competence and intra-species cell–cell communication. Ultimately as summarized in Figure 6, suppression of theses regulatory systems induced attenuation of the stress response and the environmental adaptation in *S. mutans*, and consequently led to declines of cell persistence and disruption of biofilm formation and integrity. The inhibition of GTFs at the transcriptional level lead to reduced production of EPS and IPS. Lack of EPS disrupts both adherence of bacteria and the structure of biofilm. When exogenous substrate is depleted, *S. mutans* can metabolize IPS [50]. Therefore, malfunction of GTFs not only directly disrupt the biofilm integrity, but also may enhance the starvation stress of *S. mutans* due to reduced preservation of IPS. Inhibition of F_1_F_0_-ATPase and small holes on the cytomembrane may directly lead to cytoplasmic acidity, which may also inhibit the normal process of glycolysis. This will in turn diminish the ATP pool, supress the activity of the proton translocator (F_1_F_0_-ATPase) and further exacerbate cytoplasmic acidity. When LDH catalyses the conversion of pyruvate to lactate under anaerobic condition, it also converts NADH to NAD+. Thus, the inhibition of LDH would also lead to increase in NADH. If not regulated by NADH oxidase and superoxide dismutase in time, for instance under excess oxidative stress, these excessive reducing equivalents will have toxic effects on *S. mutans* [51,52]. In other words, a series of cascaded biological effects at molecular levels may be triggered by GH12 at 1/2 MIC in cells of *S. mutans*, which thereby leads to reduced acidogenicity, compromised aciduricity, declined EPS synthesis and defective ability to from biofilms.

Previous studies have provided evidence that antimicrobial agents at subinhibitory concentrations may induce alternations in bacterial properties, including changes in morphology and ultrastructure [53], as well as inhibition or stimulation of virulence factors [54–56]. In our study, 1/4 MIC and 1/8 MIC of GH12 could slightly enhance acid production, and cause different changes in acid tolerance and EPS production (Figures 2–4). 1/8 MIC of GH12 promoted the gene expression of *ldh, atpD, gtfC, gtfD, vicR*, and *liaR* (Figure 5). In particular, it is noteworthy that 1/8 MIC of GH12 significantly accelerated the process of pH drop, and slightly lowered the terminal pH (Figure 2(a)), but lactic acid production and enzymatic activity of LDH were not significantly affected by GH12 at 1/8 MIC, indicating that GH12 at subinhibitory levels may also influence other glycolytic enzymes not recognized yet, such as the carbohydrate phosphotransferase systems [57], which need further studies. Moreover, Senadheera et al. showed that inactivation of VicRK induced impaired acid production [58], so we assumed there might be a correlation between upregulated *vicR* by GH12 at 1/8 MIC and accelerated pH drop, which also needs to be further investigated.

However, beyond the threshold of 1/2 MIC (4 mg/L), all virulence factors of *S. mutans* were inhibited by GH12. This saltatorial pattern has also been observed when *S. mutans* was treated by hydrogen peroxide, methyl viologen and chlorhexidine in studies of Bitoun et al. [59]. Thus, for efficiently inhibiting cariogenic virulence of *S. mutans*, concentration of GH12 should be maintained not lower than 1/2 MIC.

In summary, based on our data, GH12 shows a potential to act as an alternative anticaries agent because GH12 at 1/2 MIC (4 mg/L) inhibited various cariogenic virulence factors of *S. mutans in vitro*, resulting in reduced acidogenicity, compromised aciduricity, declined EPS synthesis and defective ability to form biofilms through reducing the activity of related enzymes, downregulating virulence genes, and inactivating specific regulatory systems. Considering the complexity of gene regulation in *S. mutans*, further studies with transcriptomic and proteomic approaches should be carried out.
